# On the Treatment of Prolapsus Ani in Infants

**Published:** 1877-04

**Authors:** Patrick Jamison

**Affiliations:** L.R.C.S. Edin.


On the Treatment of Prolapsus Ani in Infants.—*By Patrick Jamison, M.A., L.R.C.S. Edin.*—Prolapsus ani in very young infants is a fairly manageable ailment, though troublesome to treat, inasmuch as its recurrence may be expected at each act of defecation. Should, however, a young mother or inexperienced nurse fail to recognise the importance of the affection, and allow the intestine to remain prolapsed for any considerable time, the case assumes much graver proportions. A larger amount of gut becomes implicated, and the surgeon experiences greater difficulty in maintaining the reduction when he has succeeded in effecting it.

The expedient which I adopted in the following case may be thought worthy of record.

I was informed that a protrusion of the bowel to the extent of two inches had existed for three weeks; during that time, the mother assured me the protrusion had only been "up" twice, and each time but for five minutes. Three days before I was called the prolapsus had increased, and when I saw it the protrusion measured four inches; was curved and conical, and of a rich red color. The child was a female, fifteen weeks old, well nourished, but its appearance was pitiable to behold. The free margins of the eyelids were red with weeping, the conjunctivæ were bleared and injected, while the irides were clear and bright. The tenesmus was almost constant.

The child had been reared by the bottle, and whenever the teat was offered it hastily and greedily seized it, and sucked as if ravenous with hunger, then suddenly and rapidly did it fling, as it were, the teat from its mouth, and burst into a cry that told full surely the terrible agony it was enduring.

Healthy fæces were freely passed during my visit. On attempting to reduce the prolapse I was met with a resistance equal to several pounds in weight, but by watching the recession of the tenesmus I was enabled to effect complete reduction. No sooner was this accomplished than a stronger effort than ordinary on the part of the child flung out the bowel into its original position. Again I replaced the protrusion, and applied firm pressure across the nates—still unavailingly. The tenesmus overcame every barrier presented, and hurled out the bowel, which recurred to its abnormal position, like a compressed caoutchouc ball resuming its original shape.

On the next occasion I followed the inverted gut with my index finger within the internal spincter, and still finding vigorous pressure, I carried up my finger until it was entirely within the rectum, the knuckles resting on the perineum, and the tip of the digit lying on the left iliac synchondrosis. Here I felt the finger tip grasped by the circular fibres of the bowel, and concluded I had reached the point of invagination. The idea then struck me that if I could continue the use of this organic and sentient bougie long enough to permit the muscular fibres to contract *in situ*, I might hope for the permanent reduction of the prolapse.

Instructing the mother what to do, I rapidly exchanged my finger for hers, and placing the infant on her knee, directed her to maintain the same position for four hours. This she faithfully did; and at the end of that time was rewarded by finding the tenesmus had completly abated, and the bowel remained within the pelvis.

Previous to leaving the child I gave it six minims of liquor morphiæ hydrochloratis, with instructions to the mother to repeat the dose every hour, so long as she felt much pressure on her finger, or if the infant cried bitterly. The mother subsequently informed me that the pressure in the course of half an hour almost ceased, and at the end of four hours her finger, she said, somehow slipped out.

During the succeeding twenty-four hours the bowel protruded slightly five times, but the mother, on the alert, promptly reduced it.

The treatment thus maintained complete reduction of the invaginated portion of the gut; a position of safety not equally assured by the application of external pads.

When I saw the infant on the second day subsequent to the operation the change of facial expression was very striking. The look of anguish had disappeared; the conjunctivæ were clear and limpid; and the child applied itself to its aliment easily and naturally. A frequent cry remained —the shrill, bitter scream due to tormina. The anguished wail which marked the agony produced by invagination and its accompanying tenesmus was gone.—*Obs. Journal.*

